# Family Conflict Associated With Intrinsic Hippocampal-OFC Connectivity in Adolescent Depressive Disorder

**DOI:** 10.3389/fpsyt.2021.797898

**Published:** 2022-01-14

**Authors:** Ruohan Feng, Weijie Bao, Lihua Zhuo, Yingxue Gao, Hongchao Yao, Yang Li, Lijun Liang, Kaili Liang, Ming Zhou, Lianqing Zhang, Guoping Huang, Xiaoqi Huang

**Affiliations:** ^1^Department of Radiology, Huaxi Magnetic Resonance Research Center, Functional and Molecular Imaging Key Laboratory of Sichuan Province, West China Hospital, Sichuan University, Chengdu, China; ^2^Department of Radiology, Sichuan Mental Health Center, The Third Hospital of Mianyang, Mianyang, China; ^3^Department of Psychiatry, Sichuan Mental Health Center, The Third Hospital of Mianyang, Mianyang, China; ^4^Research Unit of Psychoradiology, Chinese Academy of Medical Sciences, Chengdu, China

**Keywords:** major depressive disorder, functional connectivity, hippocampus, adolescent, family conflict

## Abstract

**Background:**

Family environment and life events have long been suggested to be associated with adolescent depression. The hippocampus plays a crucial role in the neural mechanism of major depressive disorder (MDD) through memory during stressful events. However, few studies have explored the exact neural mechanisms underlying these associations. Thus, the current study aimed to explore alterations in hippocampal functional connectivity (FC) in adolescent MDD based on resting-state functional magnetic resonance imaging and further investigate the relationship between hippocampal FC, environmental factors, and clinical symptom severity.

**Methods:**

Hippocampal FC was calculated using the seed-based approach with the bilateral hippocampus as the seed for 111 adolescents with and without MDD; comparisons were made between participants with MDD and controls. We applied the Chinese version of the Family Environment Scale (FES-CV) and Adolescents Self-Rating Life Events Checklist (ASLEC) to evaluate family environment and life stress. Their relationship with hippocampal FC alterations was also investigated.

**Results:**

We found that compared to controls, adolescents with MDD showed decreased connectivity between the left hippocampus and bilateral orbital frontal cortex (OFC) and right inferior temporal gyrus. In addition, the hippocampal-OFC connectivity was negatively correlated with conflict scores of the FES-CV in the MDD group and mediated the association between family conflict and depressive and anxiety symptoms.

**Conclusion:**

Our findings are novel in the field and demonstrate how family conflict contributes to MDD symptomatology through hippocampal-OFC connectivity; these findings may provide potential targets for personalized treatment strategies.

## Introduction

Adolescence is a critical period of brain development and neurological and cognitive maturation (1) and is regarded as a time of “storm and stress” (2). The brain is more susceptible to the effects of environmental stress at this particular stage (3, 4). There is a notable incidence of major depressive disorder (MDD) during adolescence, which may lead to chronicity throughout life with high recurrence rates (5, 6). MDD leads to serious social and educational impairments and is closely associated with suicide (7). The developmental trajectory of depression appears to start with some environmental risk factors, such as early-life adversities, and occurs as a result of abnormalities in the brain (8).

Family environment and life stress events are both risk factors for adolescent MDD (9–12) and may also affect brain development structurally and functionally (13–15), particularly the hippocampus (16–18). For example, smaller hippocampal volume partially mediated the effect of early-life adversity on depressive episodes from a longitudinal study (19). The hippocampal network can modulate the feeling of stress (20) and play an important role in memory (21, 22). And stress can influence memory performance through hippocampal functional connectivity (FC) on a systems level (23). In recent years, several studies explored the resting-state FC (RSFC) alterations in adult MDD with bilateral hippocampus and hippocampal subfields as selected seeds, showing a significantly decreased RSFC between the bilateral hippocampal and prefrontal regions, insula, bilateral limbic system, subcortical areas, temporal lobe, and cerebellum (24–31).

Although many studies have detected altered hippocampal FC in adult MDD, only two studies with small sample sizes have investigated hippocampal FC changes in adolescent MDD. One study reported decreased intrinsic connectivity between the right hippocampus and the right insula and right middle frontal gyrus (32) in adolescents with depression comorbid with obsessive-compulsive disorder and other anxiety disorders. Another study showed significant hypoconnectivity between the bilateral hippocampus and prefrontal cortex (PFC) regions based on the region of interest (ROI)-to-ROI technique and excluded the exploration of hippocampal connectivity in other brain regions (33). However, no study has explored the relationship between hippocampal FC and stress events and depressive symptoms.

Thus, in the current study, we aimed to investigate the alteration of hippocampal FC on the whole brain base and further explore its relationship with family environment and life events in adolescent MDD by recruiting a relatively large sample of drug-naïve patients with no comorbidities to exclude the confounding effects of medication and comorbidities in the current study. We hypothesized that there are abnormalities in the intrinsic hippocampal function in emotional-related networks in adolescents with depression, and these abnormalities are related to the risk factors and symptom severity of adolescent MDD.

## Methods

### Participants

Sixty-eight first-episode and medication-naïve patients with MDD were recruited from The Third People's Hospital of Mianyang, Sichuan, China. All patients were diagnosed by two professional child and adolescent psychiatrists (Y. Li and G. Huang). The inclusion criteria were as follows: (1) age between 12 and 18 years; (2) Hamilton Depression Scale (HAMD) score ≥8; (3) no history of drug therapy and psychotherapy; and (4) no comorbid psychosis disorder (e.g., bipolar disorder, attention-deficit/hyperactivity disorder, autism, and eating disorder) and family history of psychosis disorders.

Forty-four healthy adolescent volunteers in the same age range were also recruited through poster advertisements from the same social demographic environment. Healthy subjects were screened using the non-patient edition of SCID to exclude any DSM-5 disorders. We also excluded healthy subjects if they had any physical disease or neurological disease, psychosis disorder, or family history of psychosis disorders. Additional exclusion for all individuals included the following: had any substance abuse and dependence and any contraindications for undergoing a magnetic resonance imaging (MRI) scan.

This study was approved by the Ethics Committee of the Third People's Hospital of Mianyang. All subjects were informed of the purpose and method of this experiment, and written informed consent was obtained from all adolescents and their patients or guardians.

### Clinical Measures

The 24-item HAMD (HAMD-24) (34) and 14-item Hamilton Anxiety Scale (35) (HAMA-14) were used to assess the severity of symptoms of depression and anxiety in all subjects. The higher the HAMD or HAMA scores, the more severe the symptoms.

The family environment was assessed using the Chinese version of the Family Environment Scale (FES-CV) (36), which includes 10 dimensions (cohesion, emotional expression, conflict, independence, achievement orientation, intellectual–cultural orientation, active–recreational orientation, moral–religious emphasis, organization, and control) with nine items for each dimension.

In addition, the frequency of stressful life events and stress response intensity was measured using the Adolescents Self-Rating Life Events Checklist (ASLEC) (37). This scale consists of six dimensions, namely, interpersonal relationships, study pressure, punishment, sense of loss, healthy adaptation, and other factors. A higher score indicates greater stress.

### MRI Data Acquisition

All subjects were scanned using a 3.0-T MRI system (Skyra, Siemens) with a 20-channel phased-array head coil. During the entire scanning procedure, subjects were instructed to relax with their eyes closed without falling asleep and without directed thoughts. T1-weighted anatomical images were scanned with the following scanning parameters: 176 slices, slice thickness = 1 mm, flip angle = 9°, matrix size = 256 × 256, TR = 1,900 ms, TE = 2.25 ms, voxel size = 1 mm × 1 mm × 1 mm.

Whole-brain resting-state functional MRI (rs-fMRI) data depicting blood oxygen level-dependent contrast were obtained using a gradient-echo echo-planar imaging sequence with the following parameters: 35 axial slices, slice thickness = 4 mm, slice gap = 0.2 mm, repetition time (TR) = 2,000 ms, echo time (TE) = 30 ms, flip angle = 90°, matrix size = 64 × 64, voxel size = 3.75 × 3.75 × 4 mm^3^, field of view (FOV) = 240 × 240 mm^2^. The rs-fMRI lasted 8 min in total, and 255 volumes were obtained for each participant.

### Data Preprocessing

The rs-fMRI data were preprocessed and analyzed using the Data Processing and Analysis for Brain Imaging toolkit (http://www.restfmri.net) (38) and the SPM12 (The Wellcome Department of Cognitive Neurology, London, UK, http://www.fifil.ion.ucl.ac.uk/spm/software/spm12, v6225) based on MATLAB R2018b. Specifically, the first 10 functional volumes were discarded for signal stabilization and adaptation of the subjects to the scanning surroundings. The remaining images were corrected for acquisition time intervals between slices. The images were then realigned to the first volume for motion correction. After corrections, these images were spatially normalized into the standard Montreal Neurological Institute (MNI) space, and each voxel was 3 × 3 × 3 mm^3^. We smoothed these images with an 8-mm full width at half maximum Gaussian kernel. The effects of drift or trends in fMRI were removed by a detrending analysis. We also regressed out white matter signals and cerebrospinal fluid (CSF) signals to reduce the effects of physiological noise (i.e., cardiac and respiratory fluctuations). Finally, band-pass filtering (0.01–0.08 Hz) was utilized.

To reduce the head motion effects of the functional data, we used a higher-level Friston 24-parameter model, which includes six head motion parameters, one previous time point of six head motion parameters, and 12 corresponding squared items. The mean framewise displacement (FD) was also calculated as a measure of the microscale head motion of each subject. The mean FD of each participant should be <0.2 mm; according to this criterion, one healthy control (HC) was excluded.

### Seed-Based FC Analysis

The bilateral hippocampal regions defined from the automated anatomical labeling atlas were selected as seeds. Seed-based RSFC analysis was performed using the RESTPlus software (http://restfmri.net/forum/index.php?q=rest). First, we extracted the time series for each seed. Subsequently, voxel-wise correlation analysis was conducted between each seed and all other voxels of the brain to acquire FC maps. Third, Pearson's correlation coefficients between each seed and all other voxels were converted to *z*-value images using the Fisher *r*-to-*z* transformation.

### Statistical Analysis

#### Group Comparison

The demographic and clinical differences between patients with MDD and HCs were calculated using two independent-sample *t*-tests and chi-square tests based on SPSS software, with a threshold at the *p* < 0.05 level.

Group comparison of the FC maps between MDD and HC was performed using the two-sample *t*-test in SPM12, with age, gender, and head motion as covariates [*p* < 0.005 at the voxel level and false discovery rate (FDR)-corrected *p* < 0.05 at the cluster level].

#### Correlation Analysis

We conducted partial correlation analysis to explore the association between hippocampal FC and scores of clinical symptom severity scales, including total scores of the HAMD and HAMA and environmental factors including scores of ASLEC and FES-CV with age and gender as covariates.

#### Exploratory Mediation Analysis

We further investigated the association of environmental risk factors with clinical symptoms in the whole group, considering the potential mediation effect of hippocampal connectivity identified above.

In addition, an exploratory mediation analysis was performed to investigate whether the hippocampal FC detected between groups would mediate the relationship between potential risk factors and depressive symptom severity using the simple mediation model (i.e., Model 4) of the PROCESS v3.3 macro in SPSS (39). In the mediation model, hippocampal FC was defined as the mediator variable, environmental factors as the dependent variable, and the HAMD or HAMA total score as the independent variable with age and gender being treated as nuisance variables. A bootstrapping approach with 5,000 iterations was performed to test the significance of the mediating effect. Effects with a bootstrapped 95% confidence interval (CI) that did not include zero were regarded as significant.

## Results

### Demographics and Clinical Characteristics

The demographic and clinical characteristics of all subjects are presented in Table 1. Compared to the HC group, the MDD group showed significantly higher HAMD and HAMA scores, conflict scores, and achievement orientation scores (*p* < 0.05). The ASLEC scores were also significantly higher in the MDD group than in the HC group (*p* < 0.05).

**Table 1:** Demographic and clinical variables in patients with MDD and HC subjects.

**Clinical data**	**MDD (*n* = 68)**	**HC (*n* = 43)**	**Statistics**	***P*-value**
Age (years)	14.634 ± 1.52	14.67 ± 1.86	−0.130	0.897
Gender (F/M)	50/18	25/18	2.847	0.092
Handedness (R/L)	67/1	41/2	1.013	0.314
Education (years)	8.51 ± 1.50	8.81 ± 1.88	−0.881	0.381
HAMD-24 total score	23.41 ± 7.02	2.07 ± 1.81	23.852	<0.001
HAMA-14 total score	18.99 ± 6.02	1.30 ± 1.57	23.034	<0.001
**ASLEC**				
Interpersonal relationship	15.72 ± 4.14	7.09 ± 4.09	10.740	<0.001
Study pressure	13.90 ± 3.88	7.93 ± 4.74	7.238	<0.001
Punishment	15.79 ± 6.28	5.00 ± 4.27	10.776	<0.001
Sense of loss	5.85 ± 2.98	2.70 ± 2.80	5.528	<0.001
Health adaptation	8.35 ± 2.70	2.58 ± 2.75	10.900	<0.001
**FES-CV**				
Cohesion	5.32 ± 3.23	5.14 ± 2.37	0.345	0.730
Emotional expression	4.25 ± 2.15	4.30 ± 1.81	−0.132	0.895
Conflict	5.07 ± 2.30	3.33 ± 1.88	4.172	<0.001
Independence	4.24 ± 1.76	4.26 ± 1.66	−0.061	0.951
Achievement orientation	6.19 ± 2.62	4.98 ± 1.64	3.002	0.003
Intellectual–cultural orientation	4.01 ± 2.51	4.72 ± 1.72	−1.758	0.082
Active–recreational orientation	4.31 ± 2.30	4.95 ± 1.91	−1.529	0.129
Moral–religious emphasis	5.09 ± 2.14	4.95 ± 1.83	0.341	0.734
Organization	5.35 ± 2.69	4.65 ± 1.63	1.710	0.090
Control	4.15 ± 2.31	3.95 ± 1.66	0.512	0.610

*MDD, major depressive disorder; HC, healthy controls; HAMD, Hamilton Depression Rating Scale; HAMA, Hamilton Anxiety Rating Scale*.

### Hippocampal RSFC Pattern

**Figure 1:**
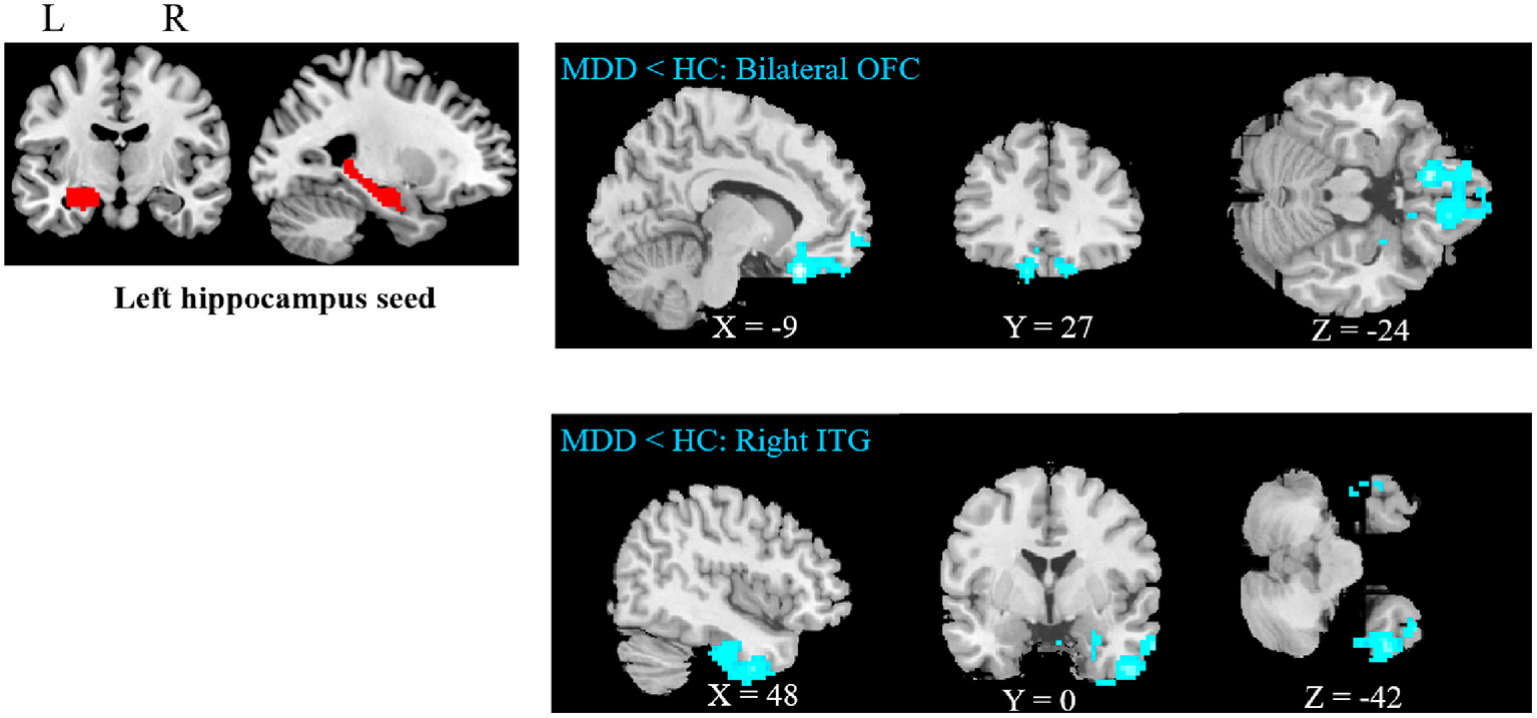
Abnormal resting-state FC between hippocampus and regions. The OFC and ITG regions showed decreased FC in the adolescent depression group. The results were obtained using a seed-based analysis method with the left hippocampus as the seed (OFC, orbital frontal cortex; ITG, inferior temporal gyrus; HC, healthy control; MDD, major depressive disorder).

Compared to HCs, adolescent MDD patients showed decreased FC between the left hippocampus and the bilateral OFC as well as between the left hippocampus and the right inferior temporal gyrus (ITG) (Figure 1 and Table 2). No significant increase in hippocampal FC was observed in the MDD group compared to the HC group. There were no significant group differences between the MDD and HC groups in the right hippocampal FC.

**Table 2:** Region of abnormal resting-state FC between the hippocampus and regions.

**Seed**	**Regions**	**Peak (MNI)**			**Voxels**	***T*-value**	***P*-value (FDR-corrected)**
		**x**	**y**	**z**			
HC > MDD							
Left	OFC	−9	27	−24	406	4.68	0.009
Hippocampus	Right ITG	48	0	−42	548	4.00	0.002

*MDD, major depressive disorder; HC, healthy controls; MNI, Montreal Neurological Institute; FDR, false discovery rate; ITG, inferior temporal gyrus; OFC, orbital frontal cortex*.

### Correlation Analysis

**Figure 2:**
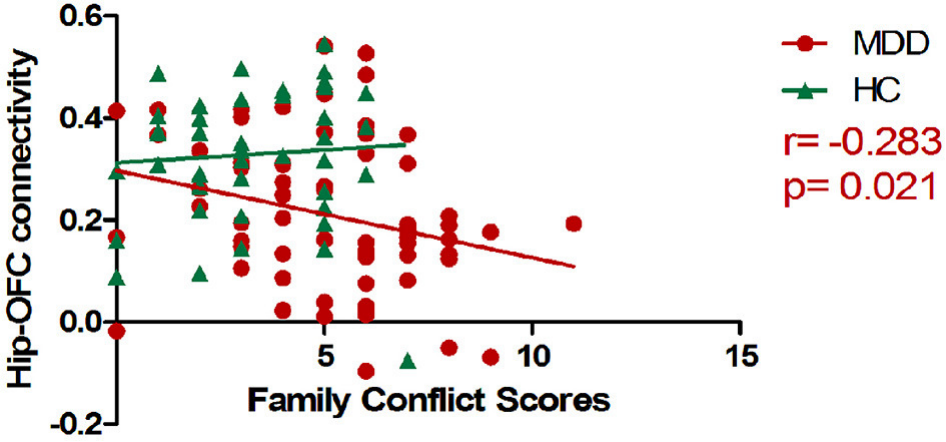
The scatter plots of the correlations between mean hippocampal-OFC functional connectivity and family conflict scores. The RSFC between the left hippocampus and bilateral OFC was negatively associated with the conflict scores of FES-CV in the MDD group (OFC, orbital frontal cortex; FES-CV, Chinese version of the Family Environment Scale; Hip, Hippocampal; HC, healthy control; MDD, major depressive disorder).

There was a negative correlation between hippocampal-OFC connectivity and family conflict scores of FES-CV in the MDD group (*p* = 0.021) after controlling for the effects of sex and age (Figure 2). No significant association between cerebral connectivity and other factors of FES-CV and ASLEC scores and clinical severity (i.e., HAMA and HAMD) were detected.

### Exploratory Mediation Analysis

**Figure 3:**
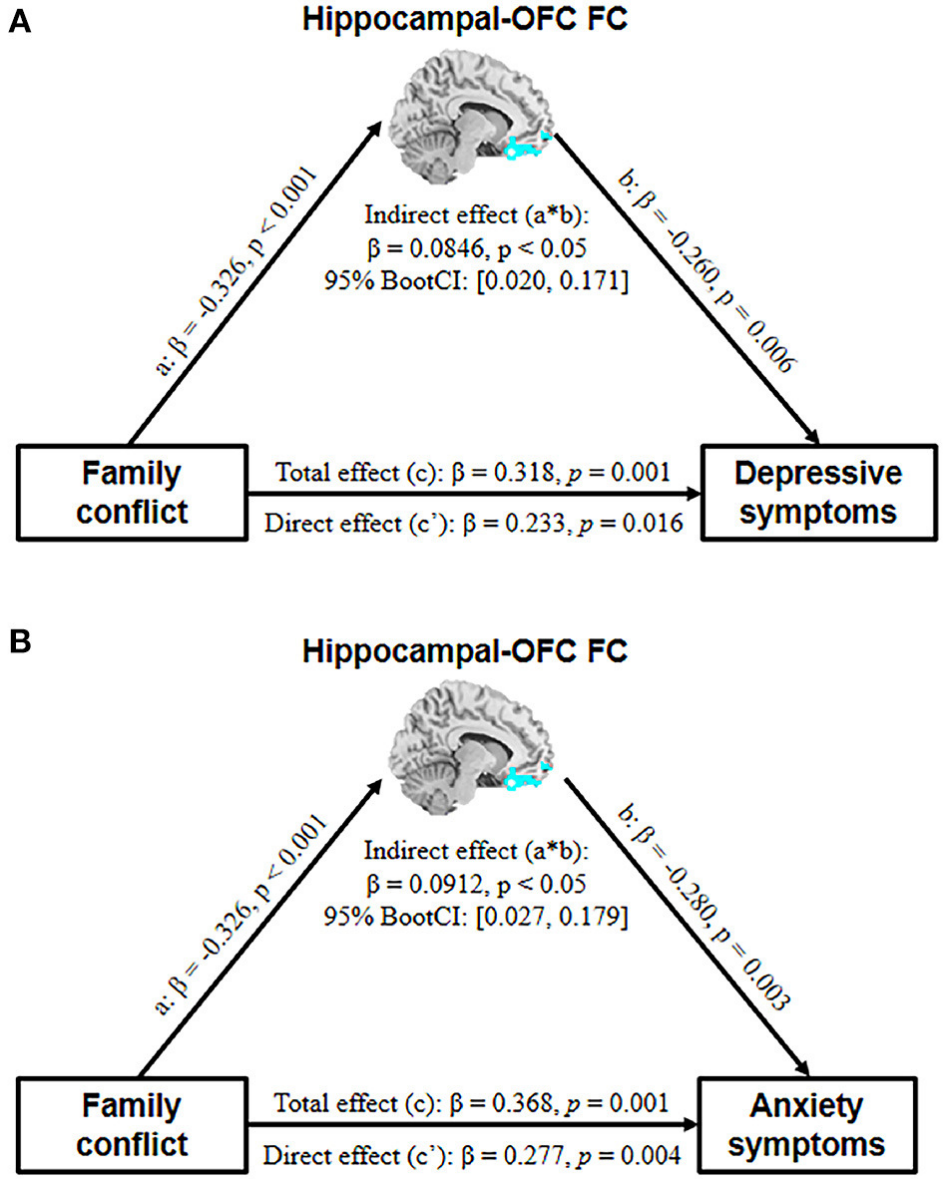
The mediation model depicts the relationship between family conflict, adolescent depressive **(A)** and anxiety **(B)** symptoms, and hippocampal-OFC FC with age and gender as covariates (OFC, orbital frontal cortex; FC, functional connectivity).

The correlations between the family conflict score and HAMD/HAMA total scores were significant in all subjects. The mediation analysis revealed that the hippocampal-OFC FC significantly mediated the association between family conflict and symptoms of depression (indirect effect = 0.0846, 95% CI = [0.020, 0.171], *p* < 0.05) and anxiety (indirect effect = 0.0912, 95% CI = [0.027, 0.179], *p* < 0.05) (Figure 3).

## Discussion

To the best of our knowledge, this is the first study to explore the relationship between hippocampal FC and environmental risk factors in adolescents with depression. Compared to HCs, adolescent MDD demonstrated significantly decreased hippocampal FC with bilateral OFC and right ITG. In addition, we found that depressed adolescents were associated with higher levels of stressful events and family conflict. However, only family conflict scores were negatively correlated with the hippocampal-OFC connectivity. More importantly, hippocampal-OFC connectivity mediated the association between family conflict and both depressive and anxiety symptoms. Our results suggest that family conflict may contribute to depressive symptoms in adolescents through changes in hippocampal-OFC connectivity.

Family conflict refers to active opposition between family members and can take a wide variety of forms, including verbal, physical, sexual, financial, and psychological. Conflicts may involve different combinations of family members: conflict within the couple or between parents and children or, again, between siblings (40). It can cause maladjustment by an adolescents' increasing emotional insecurity about the family system (41, 42) and affect children's levels of resilience, such as low self-esteem, mental fatigue, anxiety, poor school performance, introvertism, depression, and self-criticism (43). The higher family conflict scores of FES-CV in adolescent MDD suggest that family members express their anger, aggression, and contradiction toward each other more openly (44).

Many studies have shown that children and adolescents with depression express higher levels of family conflict than do HCs (45–48). Children with a family history of depression are at an increased risk of developing depressive symptoms in response to family conflicts (49). More importantly, it can be a stressful event for adolescents and increase the risk of depression (50–52). We speculate that irritability, a core symptom of adolescent depression, may be related to a high-family-conflict environment. A developmental model of depression based on vulnerability diathesis and stressful life showed that early adverse events foster negative attitudes and biases about the self, which can be activated by later adverse events impinging on the specific cognitive vulnerability and lead to depression (53).

Many researchers have demonstrated that the hippocampus and OFC work together to mediate responses to stressful experiences (54, 55) and are associated with impaired cognition in depression (56, 57). Hippocampus and OFC both have been proposed to encode parallel but interactive “cognitive maps” that capture complicated relationships between various kinds of information from the environment (58). Cognitive maps provide useful scaffolds for planning complex behaviors and thus can promote model-based learning and behavior (59). A previous task-based fMRI study (60) suggested that increased hippocampal-OFC connectivity could facilitate model-based interference. Therefore, the decreased hippocampal-OFC observed in this study might be linked to abnormalities in processing of information from the external environment and inferring future outcomes, thus leading to cognitive impairments in adolescents with MDD.

In addition, family conflict can also serve as childhood early-life stress, which may independently predict adulthood MDD diagnosis and be associated with smaller volumes of the OFC and left hippocampus (52). Previous research has shown diminished connectivity between the hippocampus and OFC during conflict resolution, a way of presenting family conflict, based on theta band coherence (61). Our finding of the mediation effect of hippocampal-OFC connectivity provides solid evidence for the involvement of these two structures in depression neuropathology. It delineated how environmental risk factors, such as family conflict, lead to depressive symptoms.

We also found that hippocampal-OFC connectivity could mediate the association between family conflict and anxiety symptoms. Stressful family environments play an important role in developing anxiety symptoms (62, 63). Hippocampal connectivity can predict the subjective feeling of stress (20). OFC dysfunction is related to failure of inappropriate fear and anxiety response inhibition (64). Taken together, our findings suggest that the interaction between the hippocampus and OFC plays a critical role in affective symptom development.

In addition, we found decreased connectivity between the left hippocampus and the right ITG in adolescent MDD. Previous studies focused on Sjogren's syndrome (65) and subcortical vascular mild cognitive impairment with depression symptoms (66) have revealed decreased connectivity between the hippocampus and ITG, which is related to cognitive impairment (such as visual memory) and depression symptoms. The interaction between the hippocampus and ITG contributes to visual memory and associative memory (67–69). Therefore, the decreased FC of the left hippocampus and right ITG in adolescent MDD may be related to impaired visual memory in this population. However, this hypothesis has yet to be elucidated in further research.

Despite this being a large, well-characterized sample, this study also has some limitations. Although our study found a close association between family conflict, hippocampal-OFC connectivity, and depressive symptoms, the result could not survive FDR correction for multiple comparisons and was limited by the cross-sectional design. Future studies should verify this result from a longitudinal perspective to identify the developmental effects of family conflict exposure on the hippocampus and OFC and further uncover the mechanisms underlying the development of depression. In addition, other environmental stress factors (e.g., child maltreatment, homelessness, and poverty) that were not included in this study were also reported to be associated with depression (8). The relationship between these environmental factors and biological markers in adolescent depression should be further investigated to facilitate the detection of individuals at risk of developing depression.

In summary, we are the first to report that family conflict may contribute to depressive symptoms in adolescents through abnormal hippocampal-OFC FC. These results provide a pathogenesis mechanism for depressive disorder in adolescents and environmental factors that may be targets for future preventive strategies.

## Data Availability Statement

The raw data supporting the conclusions of this article will be made available by the authors, without undue reservation.

## Ethics Statement

The studies involving human participants were reviewed and approved by the Ethics Committee of the Third People's Hospital of Mianyang. Written informed consent to participate in this study was provided by the participants' legal guardian/next of kin.

## Author Contributions

RF, MZ, LZha, and XH designed the study. RF, YL, LL, HY, LZhu, and GH participated in the patient recruitment. WB performed the MRI preprocessing and quality assessment. RF, WB, YG, and KL performed the data analyses and statistics. RF and WB wrote the article. XH, YG, and KL revised it critically for important intellectual content. All authors approved the final version to be published.

## Funding

This study was supported by grants from National Natural Science Foundation of China (No. 81671669), Sichuan Provincial Youth Grant (No. 2017JQ0001), 1.3.5 project for disciplines of excellence, West China Hospital, Sichuan University (No. ZYJC21041), and Clinical and Translational Research Fund of Chinese Academy of Medical Sciences (No. 2021-I2M-C&T-B-097).

## Conflict of Interest

The authors declare that the research was conducted in the absence of any commercial or financial relationships that could be construed as a potential conflict of interest.

## Publisher's Note

All claims expressed in this article are solely those of the authors and do not necessarily represent those of their affiliated organizations, or those of the publisher, the editors and the reviewers. Any product that may be evaluated in this article, or claim that may be made by its manufacturer, is not guaranteed or endorsed by the publisher.
